# Cadmium Exposure Affects Serum Metabolites and Proteins in the Male Guizhou Black Goat

**DOI:** 10.3390/ani13172705

**Published:** 2023-08-25

**Authors:** Yuanfeng Li, Xiaoyun Shen

**Affiliations:** 1 College of Life Science and Engineering, Southwest University of Science and Technology, Mianyang 621010, China; liyf@mails.swust.edu.cn; 2 State Key Laboratory of Sheep Genetic Improvement and Healthy Production, Xinjiang Academy of Agricultural and Reclamation Sciences, Shihezi 832000, China

**Keywords:** metabolomics, proteomics, heavy metal, heavy metal exposure, antioxidant capacity, goat meat

## Abstract

**Simple Summary:**

Cadmium (Cd), a toxic heavy metal, can extensively pollute the environment after long-term accumulation in the natural environment. After Cd is absorbed by the human body through the food chain, it can also bring great harm to human health. In this study, goats were taken as the research object to study the key pathways and potential therapeutic targets of Cd metabolism to ultimately provide technical support for the prevention and treatment of Cd exposure.

**Abstract:**

Food safety and environmental pollution are the hotspots of general concern globally. Notably, long-term accumulation of trace toxic heavy metals, such as cadmium (Cd), in animals may endanger human health via the food chain. The mechanism of Cd toxicity in the goat, a popular farmed animal, has not been extensively investigated to date. Therefore, in this study, ten male goats (Nubian black goat × native black goat) were exposed to Cd via drinking water containing CdCl_2_ (20 mg Cd·kg^−1^·BW) for 30 days (five male goats per group). In this study, we used an integrated approach combining proteomics and metabolomics to profile proteins and metabolites in the serum of Cd-exposed goats. It was found that Cd exposure impacted the levels of 30 serum metabolites and 108 proteins. The combined proteomic and metabolomic analysis revealed that Cd exposure affected arginine and proline metabolism, beta-alanine metabolism, and glutathione metabolism. Further, antioxidant capacity in the serum of goats exposed to Cd was reduced. We identified CKM and spermidine as potential protein and metabolic markers, respectively, of early Cd toxicity in the goat. This study details approaches for the early diagnosis and prevention of Cd-poisoned goats.

## 1. Introduction

Goat meat is one of the main sources of food for human consumption. Food quality and safety are closely related to the human quality of life but cannot be guaranteed at all times. The increasing number of mining operations and the wide use of heavy metals under industrial production or other chemical contamination can lead to the pollution of urban domestic water [1,2]. The subsequent use of the polluted water in agriculture compounds the problem of environmental heavy-metal pollution, as heavy metals enter the ecosystem and are distributed therein via various pathways. They accumulate in animals through different methods of intake, ultimately leading to animal death and affecting human food safety.

Cadmium (Cd) is a hazardous metal with a long biological half-life and significant toxicity; it is slowly metabolized, which leads it to be easily accumulated in animals. According to the laws in China, the maximum limit standards for Cd in food are 0.10 mg·kg^−1^ for meat, 0.5 mg·kg^−1^ for animal liver, and 1.0 mg·kg^−1^ for animal kidney [3]. Long-term exposure to Cd may directly lead to pathological changes in the tissues and organs of the immune, respiratory, and digestive systems, and affect human health [4,5,6,7]. While research into the chronic accumulation of Cd and Cd poisoning intensifies, the pathogenesis is far from clear.

Heavy metals impact the cellular antioxidant system, an important homeostatic defense system in animals. Serum antioxidant enzymes, such as superoxide dismutase (SOD) and glutathione peroxidase (GSH-Px), can be used to characterize changes in the antioxidant capacity [8]. Cd induces oxidative stress by increasing the reactive oxygen species levels in cells, reducing the antioxidant capacity, and increasing the risk of infection [9]. However, changes in the enzyme activities of animal serum cannot be used, as of yet, to specifically detect Cd poisoning.

Omics technologies are widely used for disease diagnosis and treatment evaluation [10]. Among “omics”, proteomics and metabolomics have been especially helpful in the study of various toxicological mechanisms [11]. Specifically, proteomics can explore changes in protein abundance, identify potential drug therapy targets, and elucidate the underlying molecular mechanisms of toxicity and cellular response to toxicity [12]. For instance, Thompson et al. (2011) [13] used proteomics to study the mechanism of copper, lead, and zinc poisoning in oysters. On the other hand, metabolomics can be used to analyze changes in the overall metabolite content and levels in biological samples elicited by changes in the environment. This approach allows for a comprehensive qualitative and quantitative analysis of the metabolites to shed light on how animals and plants deal with and counterbalance the effect of various toxic factors at the molecular level [14]. 

Omics technologies are widely used in environmental pollutant research to directly evaluate health effects of exposure to low doses of environmental pollutants [15]. They have also been used to investigate the toxic mechanism of Cd-poisoned rat urine [16] and kidney [14]. Further, a combined application of proteome and metabolome analyses in relation to disease diagnosis has been reported [17,18,19]. These two omics techniques can be integrated to provide insight into the physiological state of an organism. One example is a study of the effect of environmental arsenic (V) on juvenile rockfish *Sebastes schlegelii*, in which these two approaches revealed several obvious toxicological effects after 14 days of exposure [20]. 

To date, few research studies on the mechanism of Cd poisoning in the goat have been reported. Accordingly, in this study, metabolomics and proteomics techniques were jointly used to investigate the changes in Cd-poisoned goat serum. We employed hydrophilic interaction liquid chromatography–ultra-high-performance liquid chromatography-Q Exactive mass spectrometry (HILIC UHPLC-QE MS) analysis and isobaric labeling based on relative and absolute quantification (tandem mass tag, TMT), accordingly. This study revealed the molecular mechanism of Cd toxicity in the goat and informs the diagnosis and biomarkers of early Cd poisoning in the goat. 

## 2. Materials and Methods 

### 2.1. Reagents and Chemicals 

Cadmium chloride (CdCl_2_) was obtained from Tianjin Zhiyuan Chemical Reagent Co., Ltd. (Tianjin, China) 

### 2.2. Animal Management and Sample Preparation

Ten healthy Guizhou black goats (Nubian black goat × native black goat, male, 10.75 ± 0.40 kg, five months) were purchased from a goat farm (Guiyang, China). Goats were kept in a sheepcote with a wooden slatted floor. The animals were fed by grazing [21] and sterilized tap water was fed to animals ad libitum. Green grass was fed to goats at 8:00 and 13:00, and concentrate supplement (200 g per goat) was supplemented at 19:00. The feed formula was displayed in Table 1. 

Goats were randomly divided into two groups with five goats per group. Based on the median lethal dose (LD_50_, 94.1 mg/kg·BW [body weight]) of Cd established previously in a study of rats orally exposed to Cd [22], CdCl_2_ (20 mg Cd·kg^−1^·BW) was provided in the drinking water of goats in the Cd group, as described in the study of Li et al. (2023) [23].

For this experiment, the daily intake of Cd for each goat was calculated according to the individual body weight (Cd: 20 mg·kg^−1^·BW), and about 5 mL of CdCl_2_ aqueous solution (57 mg/mL) was diluted to 1000 mL of aqueous solution. Each goat in the Cd group was fed 1000 mL of the diluted CdCl_2_ solution every day; animals in the control group were fed an identical amount of sterilized tap water. Following a 7-day adaptation, all animals were fed water containing Cd or no Cd, depending on the experimental group, for 1 month. After one week of Cd feeding, some goats from the experimental group exhibited clinical symptoms, such as listlessness, rapid heartbeat, reduced food intake, pale mucosa, and unstable standing.

At the end of the experiment, 10 mL of blood was collected into vacuum tubes containing heparin sodium; this was centrifugated for 10 min (3000× *g* and 4 °C). The separated plasma samples were cryopreserved in a refrigerator at −80 °C for further analysis. The experimental overview is illustrated in Figure S1.

### 2.3. Cd Level of the Goat Serum

The Cd level of goat serum was measured using a graphite furnace atomic absorption spectrophotometer (AA–7000, Shimadzu Corporation, Kyoto, Japan).

### 2.4. Antioxidant Status of the Goat Serum

The levels of SOD, GSH-Px, catalase (CAT), and malondialdehyde (MDA), and total antioxidant capacity (T-AOC) in the serum were determined using commercial test kits (Nanjing Jiancheng Bio-Engineering Institute, Nanjing, China), as specified in the manufacturer’s guidelines [24] (Li et al., 2021). 

### 2.5. Metabolomics and Proteomics Analysis

#### 2.5.1. Serum Preparation for Metabolomics and Proteomics

Serum samples were prepared according to Shen et al. (2021) [12], and the metabolites and proteins were extracted as described previously [12]. Two microliters of the supernatant were analyzed via HILIC UHPLC-QE/MS (Thermo Fisher Scientific, Waltham, MA, USA). For protein analysis, the most abundant serum proteins were first removed by passing through an Agilent multiple-affinity removal system (Thermo Fisher, Waltham, MA, USA). Then, a moderate amount of proteins was digested using trypsin [25] and desalted. Protein concentrations in the extracts were determined using the BCA method [26], and the abundance of serum proteins was determined using TMTs (Section 2.5.2). The stability of the LC-MS/MS analysis sequence was monitored by analyzing five samples for quality control from the adducted samples.

#### 2.5.2. Data Acquisition of Metabolomics and Proteomics

Chromatographic separation of serum samples and mass data acquisition were carried out using an UPLC system (Ultimate 3000, Thermo Fisher, Waltham, MA, USA) and HILIC UHPLC-QE/MS (Thermo Fisher, Waltham, MA, USA), respectively. Serum samples were injected into an ACQUITY UPLC BEH C18 column (2.1 × 100 mm, 1.7 μm) at 40 °C, and the flow rate was 0.30 mL/min. The mobile phase consisted of 0.1% formic acid in water (A) and acetonitrile (B). The mass spectrometer, equipped with a dual electrospray ionization source (ESI), operated under positive and negative ion modes (the scan time: 5 spectra/s, centroid mode: 50~1000 *m*/*z*). The linear gradient program for the LC and mass spectrum (MS) analysis was as depicted by Shen et al. (2019) [27]. 

For protein analysis, the TMT reagent (Thermo Fisher) was used to label the peptide mixture (100 μg). Then, strong cation exchange chromatography and the AKTA purifier system were used to fractionate the labeled peptides. The analytical procedure had been previously described [12].

#### 2.5.3. Multivariate Statistical Analysis and Quantitative Proteomic Analysis

The raw MS spectra of the detected metabolites were first converted into a common data format (.mzML) using Compound Discoverer software (Thermo Fisher, USA; version 3.0). These metabolites (VIP > 1, adjusted *p* < 0.05) were identified as potential biomarkers of Cd exposure. 

Trypsin-digested protein extracts were injected into a nano-LC-MS/MS system for protein analysis. The analytical procedure was previously described [12]. More details regarding the metabolomic analysis and the linear gradient program for TMT protein analysis are provided in the Supplementary File S1. 

#### 2.5.4. Identification and Bioinformatics Analysis of Metabolites and Proteins

Metabolites were confirmed using LS-MS/MS analysis. The analysis software (Compound Discoverer version 3.0, STRING software, et al.), databases (GO, KEGG, and HMDB), and the detailed parameters were described in previous studies [23]. The *p*-value for differentially abundant proteins (DAPs) was calculated using the *t*-test. DAPs with an average TMT ratio of ≥1.2 or ≤0.833 were considered to be extremely differential [28]. 

#### 2.5.5. Target Analysis of DEPs with Parallel Reaction Monitoring (PRM) 

Representative proteins were chosen for targeted quantification and verification, according to the study of Shen et al. (2019) [27]. The Skyline software (MacCoss Lab Software, version 3.5.0) was used for MS data processing. Detailed verification procedures were described in the Supplementary File S1.

#### 2.5.6. Data Processing

Data were presented as the mean of 5 biological replicates and were analyzed using the one-way analysis of variance (ANOVA) (Statistical Package for the Social Sciences, SPSS, version 23.0, Inc., Chicago, IL, USA). The significance threshold was set at *p* < 0.05.

## 3. Results 

### 3.1. Cd Level of the Goat Serum

**Figure 1:**
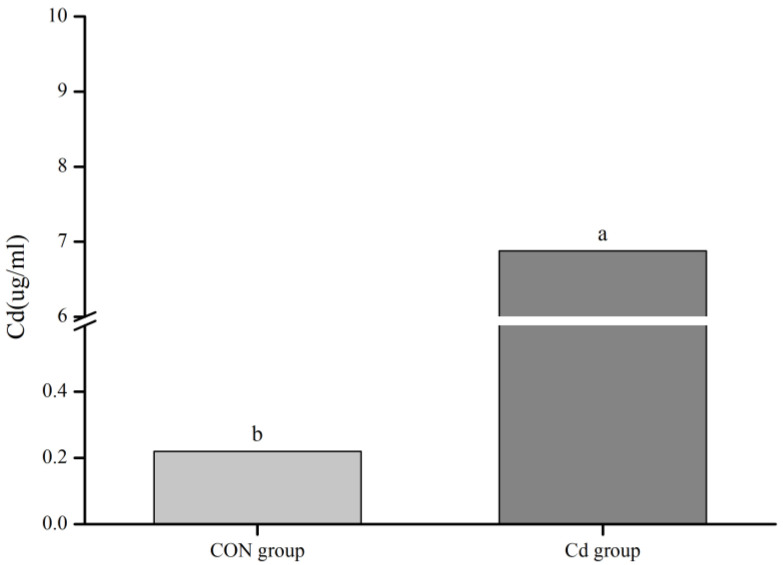
Cd level of the goat serum. Different lowercase letters indicate significant differences (*p* ˂ 0.05).

The Cd level in the serum of the Guizhou black goat from the Cd group was about 31 times higher than that from the control group (*p* < 0.05, Figure 1).

### 3.2. Antioxidant Levels of the Goat Serum

**Figure 2:**
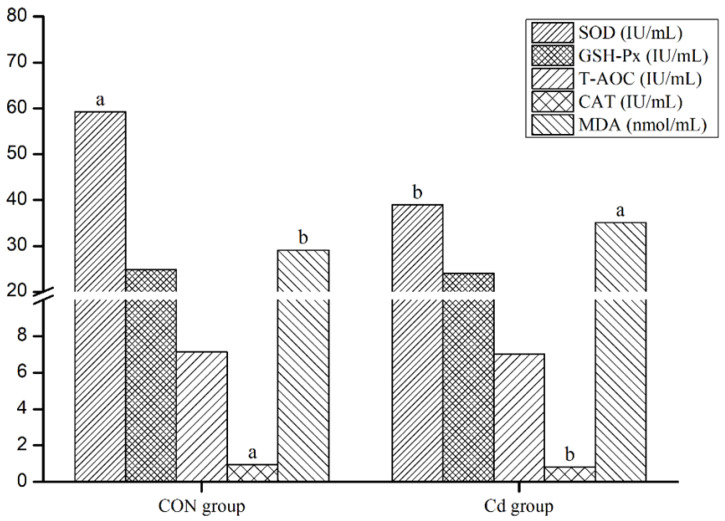
Antioxidant capacity in the goat serum. Different lowercase letters for the same measurement type for the two groups indicate a significant difference (*p* < 0.05; SPSS version 23.0; n = 5). Abbreviations: SOD, superoxide dismutase; GSH-Px, glutathione peroxidase; T-AOC, total antioxidant capacity; CAT, catalase; MDA, malondialdehyde; and CON, control.

Compared with the control group, the serum SOD and CAT activities in the Cd group were significantly decreased, while the serum MDA levels in the Cd group were significantly increased (*p* < 0.05, Figure 2).

### 3.3. Serum Metabolic Response in Goats Exposed to Cd

**Figure 3:**
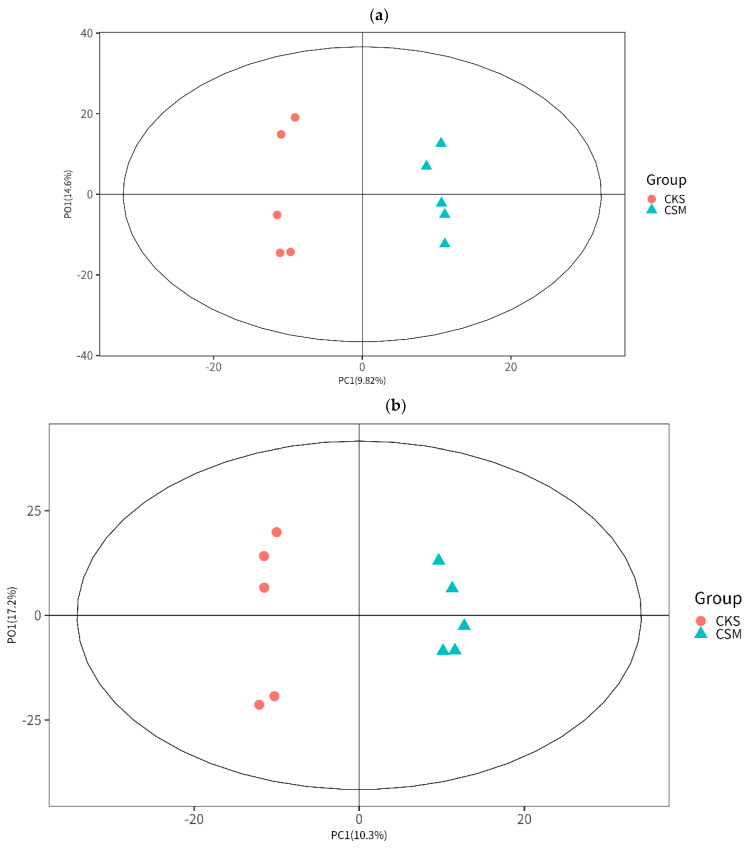

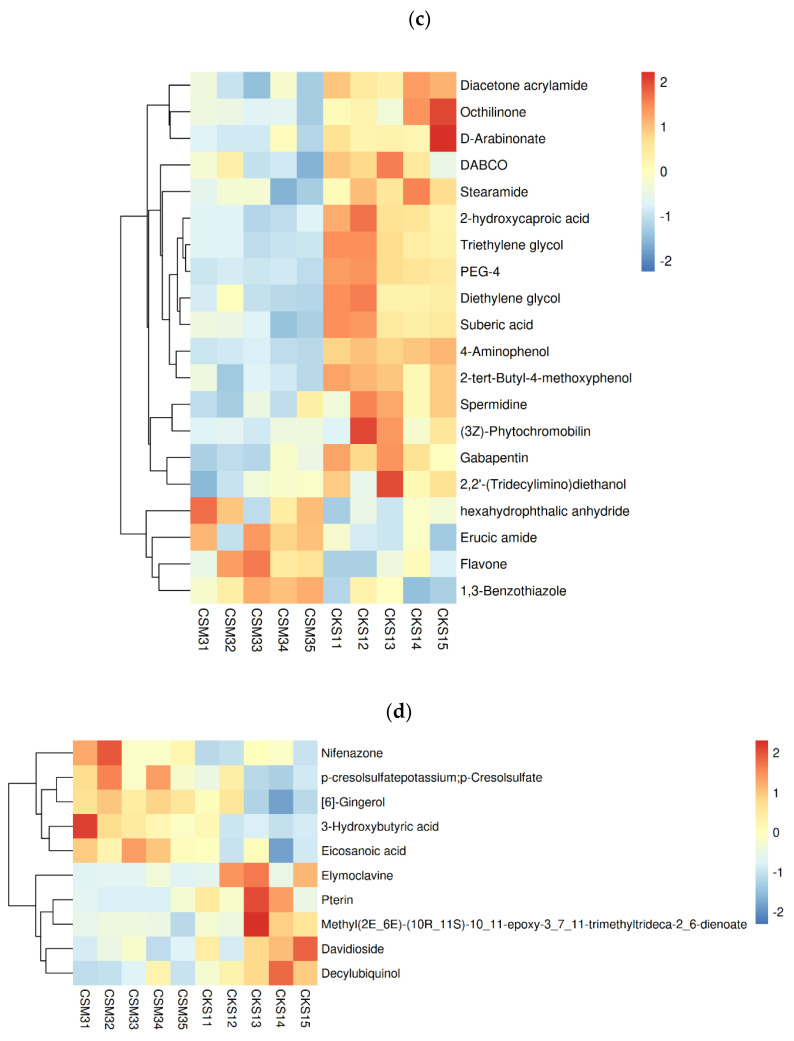
OPLS-DA score plots and unsupervised hierarchical clustering heat maps. (**a**,**b**) OPLS−DA score plots for samples analyzed via HILIC UHPLC-QE MS under positive (**a**) and negative (**b**) ion modes. Samples in the Cd group clustered separately from those in the control group in the plot. In the control group vs. Cd group comparison, ESI(+), R^2^ X = 0.244, R^2^ Y = 0.697, and Q^2^ = −0.617; meanwhile, ESI(–), R^2^ X = 0.250, R^2^ Y = 0.572, and Q^2^ = −0.511; where R^2^ X and R^2^ Y indicated the fraction of the variables explained by the model, and Q^2^ indicated the predictive ability of the model. (**c**,**d**) Unsupervised hierarchical clustering heat maps of metabolites from the goat serum obtained in the positive (**c**) and negative (**d**) ion modes. The signal intensity of related metabolites was indicated by the different colors.

Total ion chromatograms of serum samples were analyzed via HILIC UHPLC-QE MS (Thermo Fisher Scientific, Waltham, MA, USA). The chromatograms displayed that the peaks were well separated, while the peak shape was presented well, and the signal strength was strong (Figure S2). The detected metabolites were listed in Table S1. The tight clustering of the quality control samples in the principal component analysis (PCA) score plots indicated that the system was stable and reproducible (Figure S3). In the partial least-squares discriminant analysis (PLS-DA; Figure S4) and orthogonal projections to latent structures discriminant analysis (OPLS-DA; Figure 3a,b) score plots, the samples between the control group and Cd group were clearly separated, indicating that Cd exposure perturbed the serum metabolic profile in the goat.

OPLS−DA was then used to determine the variables that caused the observed separation. Thirty metabolites in the Cd group (VIP > 1, *p*-value < 0.05) were significantly differentially expressed, i.e., nine metabolites were up-regulated and twenty-one metabolites were down-regulated. The differentially expressed metabolites (DEMs) were listed in Table 2. Similar, serum samples from the control group and Cd group were separately clustered in a hierarchical clustering dendrogram (Figure 3c,d). In addition, correlation analysis revealed that metabolites with similar abundance patterns were clustered together, indicating an internal relationship among them. Cd exposure affected the levels of most metabolites.

A summary of the results of Compound Discoverer pathway analysis was shown in Table S2. Ten metabolic pathways were pronouncedly altered upon Cd exposure (Figure S5). The associated metabolites were involved in numerous pathways, such as the cAMP signaling pathway, beta-alanine metabolism, glutathione metabolism, ascorbate and aldarate metabolism, arginine and proline metabolism, ATP-binding cassette (ABC) transporters, porphyrin metabolism, and bile secretion. KEGG analysis of the corresponding metabolic pathways confirmed that the cAMP signaling pathway and beta-alanine metabolism were the main affected metabolic pathways (Table 2).

### 3.4. Serum Proteomic Response to Cd Exposure in the Goat

TMT labeling was used to investigate the serum proteome of goats. The analysis yielded 2504 spectra, resulting in the identification of 669 peptides (Table S3). Overall, basing on the presence of distinct peptides,108 DAPs were identified with 44 up-regulated proteins and 64 down-regulated proteins in the Cd group (Table 3).

**Figure 4:**
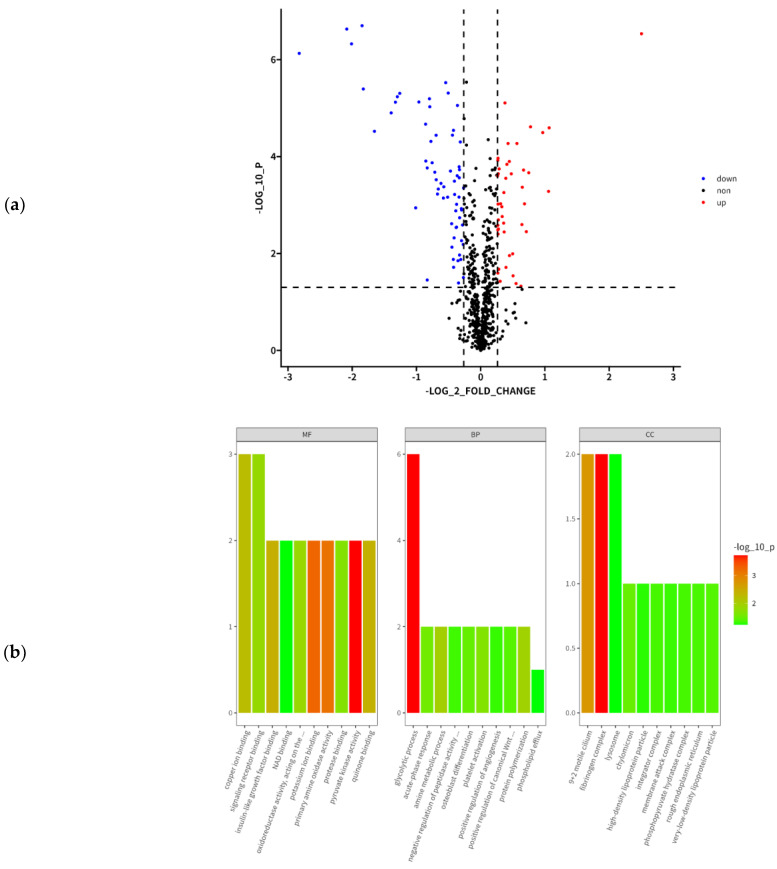

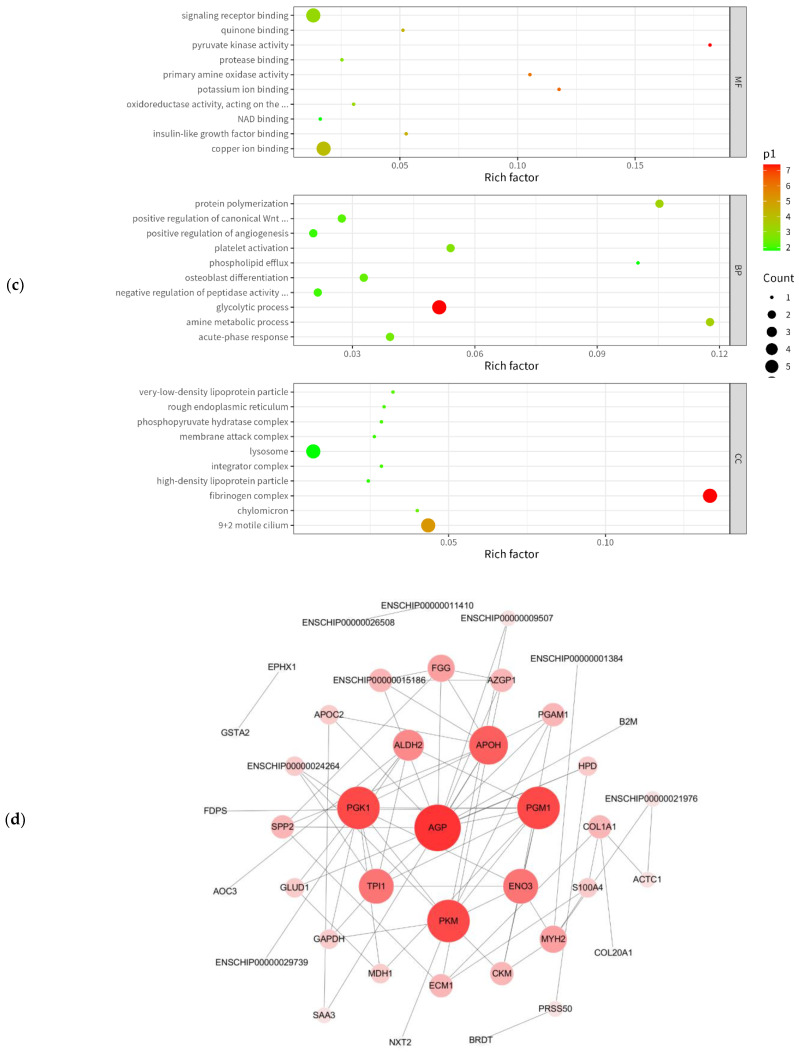
Cluster heat map, GO enrichment analysis, KEGG pathway analysis, and PPI of DEPs. (**a**) Number of DEPs in the Cd group vs. control group comparison. Gray, proteins with unchanged abundance. Red, up-regulated proteins in the Cd group. Blue, down-regulated proteins in the Cd group. (**b**) Enriched GO terms of DEPs related to the MF, BP, and CC. The top 10 enriched biological processes were shown and the number of proteins in each category was indicated. (**c**) KEGG pathway classification and functional enrichment of the predicted DEPs in the Cd group vs. control group comparison. (**d**) The PPI network of DEPs in the Cd group vs. control group comparison. The protein interaction network was plotted using Cytoscape software. Different color nodes represent the DEPs, and the circle size indicated protein connectivity, i.e., the number of proteins that directly interacted with a protein of interest.

The number of DEPs in the Cd group vs. control group were displayed in Figure 4a. To gain insight into the biological changes associated with Cd exposure, DAPs were classified into the GO categories, including CC, MF, and BP (Figure 4b). The main GO terms in each category for DAPs in the control group vs. Cd group comparison were shown in Figure S6. The identified proteins in the CC category were mostly located at the cell junction (14.29%), cell periphery (42.86%), cell projection (4.29%), cell surface (7.14%), extracellular periphery (17.86%), and others (3.57%) (Figure S6a). DEPs in the MF category were mainly related to protein binding (62.4%), transferase activity (21.6%), and others (16%) (Figure S6b). Finally, DAPs in the BP category were involved in the following processes: anatomical structure development (46.11%), establishment of localization (5.39%), organic substance metabolic process (6.99%), others (21.96%), primary metabolic process (5.59%), regulation of biological process (8.38%), and regulation of molecular function (5.59%) (Figure S6c).

GO analysis was used to obtain information on the biological background of the identified DAPs. Forty-eight KEGG pathways were identified as altered in the Cd group. The pathways enriched in that group included phenylalanine metabolism, beta-alanine metabolism, glutathione metabolism, glycolysis/gluconeogenesis, among others (Table S4). The critical pathways for Cd poisoning was phenylalanine metabolism and beta-alanine metabolism. The DEP protein–protein interaction network (Cd group vs. control group) was shown in Figure 3d.

### 3.5. PRM Verification of the Proteomics Findings

PRM was used to validate the proteomic data at the protein level. Six DAPs (amine oxidase, alpha-2-glycoprotein 1, zinc binding, Ig-like domain-containing protein, alpha-2-macroglobulin, and beta-2-microglobulin) were selected for analysis. The PRM-detected fold changes of the abundance of the selected proteins were in accordance with the results of TMT analysis (Table S5), indicating that the proteomics data were credible. These DEPs were up-regulated in the Cd group.

### 3.6. Integrated Analysis of Proteomics and Metabolomics Data

Finally, KEGG analysis was used to identify the shared pathways of the identified differential metabolites and DAPs (Figure S7a). This analysis revealed that three metabolic pathways (arginine and proline metabolism, beta-alanine metabolism, and glutathione metabolism) played an important role in Cd poisoning.

**Figure 5:**
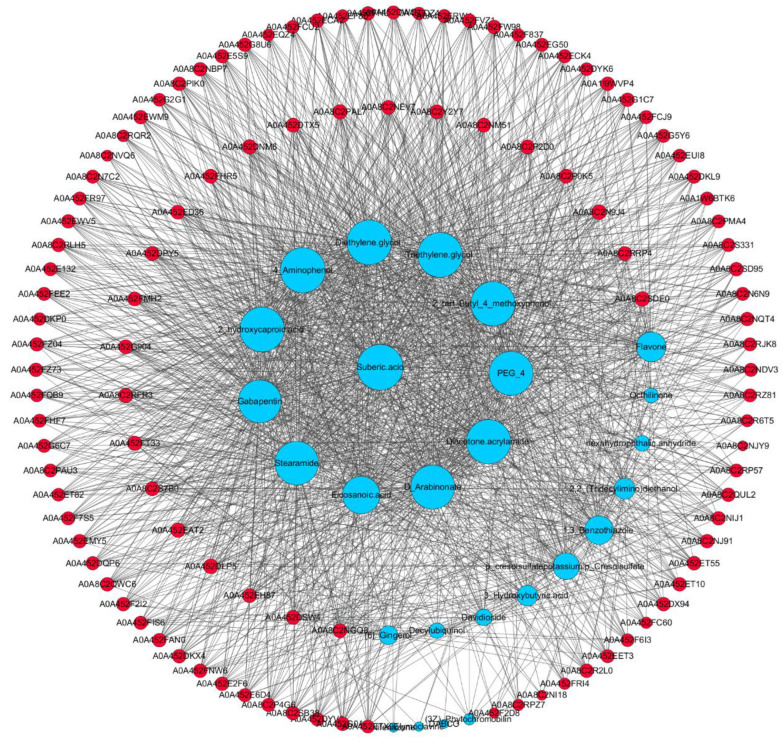
Correlated pathway analysis of proteins and metabolites. Names of differential proteins and metabolites were indicated by different colors, and the circle size indicated the degree of interaction.

The networks of the metabolic pathways that were significantly altered in response to Cd exposure were next determined and analyzed in detail (Figure 5). The analysis revealed that Cd regulated the arginine and proline metabolism, beta-alanine metabolism, and glutathione metabolism by altering the levels of spermidine. It regulated the arginine and proline metabolism by affecting the levels of *CKM*, and the beta-alanine metabolism by affecting the protein levels of primary amine oxidase. Cd regulated the glutathione metabolism by affecting the protein levels of *GSTA2*. Overall, Cd mainly improved arginine and proline metabolism, beta-alanine metabolism, and glutathione metabolism by down-regulating the levels of spermidine and *CKM*, and up-regulating the levels of primary amine oxidase (Figure 5).

## 4. Discussion

In the present study, we used metabolomics in conjunction with proteomics to explore the mechanism of Cd poisoning in the goat. Differential metabolites and proteins in control vs. Cd-exposed comparisons were identified and used to characterize the molecular mechanism of Cd poisoning. The separation of the serum samples from the control and Cd groups observed in PCA and OPLS-DA indicated that Cd poisoning affected the serum metabolomic profile. The results of metabolic pathway analysis indicated that the mechanism of Cd poisoning might be chiefly related to the cAMP signaling pathway and beta-alanine metabolism. Further, TMT-based quantitative proteomics identified 108 DAPs in the control group vs. Cd group comparison. Bioinformatics analysis revealed that the DAPs were mainly related to phenylalanine metabolism and beta-alanine metabolism. Further, PRM validated the observed changes in the abundance of amine oxidase, alpha-2-glycoprotein 1, zinc binding, Ig-like domain-containing protein, alpha-2-macroglobulin, and beta-2-microglobulin.

### 4.1. Antioxidant Capacity in the Black Goat Serum

The antioxidant system is a cellular defense system that scavenges free radicals. Accordingly, the levels of antioxidant enzymes (SOD, GSH-Px, CAT, among others) can be used to assess the antioxidant capacity in the body [29]. In Cd-treated goats, the serum SOD and CAT activities in the Cd group were significantly decreased and the serum MDA levels were significantly increased, which indicated that the antioxidant system of Cd-exposed goats was damaged. According to a previous study, long-term Cd accumulation causes general cell necrosis and can lead to tumor formation [30]. The observed changes of serum antioxidant enzyme levels might indicate that the tested Cd dose was toxic to the black goat.

### 4.2. Glycolysis/Gluconeogenesis Alteration upon Cd Exposure

In the course of glycolysis, glucose is metabolized into pyruvate and lactate. In contrast, during gluconeogenesis, simple non-sugar precursors, such as pyruvate and lactate, are converted into sugar. These pathways are crucial for glucose metabolism [31]. In the current study, nine DAPs related to glycolysis/gluconeogenesis were identified, namely, *ENO3*, *ALDOA*, *PGK1*, *PKM*, *GAPDH*, *PGM1*, *TPI1*, *CKM*, and *PGAM1*. Their abundance was down-regulated in the goats that were exposed to Cd.

*ENO3* is a metalloenzyme that plays a role in glycolysis and also inhibits tumor formation during the development of hepatocellular carcinoma [32]. As an important glycolytic enzyme, *ALDOA* is a potential prognostic biomarker and therapeutic target of lung adenocarcinoma [33]. *PGK1* is the first ATP-generating enzyme of the glycolytic pathway. It is highly expressed in a variety of tumors and is crucial for coordinating energy production with biosynthesis and redox balance [34]. *PKM* is one of the main rate-limiting enzymes of glycolysis, catalyzing the conversion of phosphoenolpyruvate and ADP into pyruvate and ATP, respectively. Its activity directly affects the speed and direction of the entire metabolic pathway [35]. *PKM2* is one of the four subtypes of mammalian pyruvate kinase that supports synthetic metabolism and is expressed in both cancer and normal tissues. *GAPDH* is another key enzyme of glycolysis. It catalyzes the first glycolytic step by converting d-glyceraldehyde-3-phosphate into 3-phosphate-d-glycerolphosphate [36]. *PGM1* is an essential enzyme of glucose metabolism. It participates in cell vitality, proliferation, and metabolism [37]. *TPI1* is a homodimeric enzyme that catalyzes the isomerization of d-glyceraldehyde-3-phosphate and dihydroxyacetone phosphate during glycolysis [38]. It has been reported that *TPI1* has an inhibitory effect on liver cancer [39]. *CKM* is mainly present in the cytoplasm and mitochondria, and is a kinase directly involved in intracellular energy transport and ATP regeneration [40]. Determination of its activity can be used for the diagnosis of skeletal muscle and myocardial diseases. Further, trace amounts of Cd inhibit the activity of *CKM* [40]. *PGAM1* is a key enzyme of sugar metabolism. It catalyzes the conversion of 3-phosphoglycerate and 2-phosphoglycerate [41]. This enzyme also generates adenosine triphosphate, stimulates anabolic pathways, maintains redox balance under hypoxic conditions, and is overexpressed in numerous cancers [41].

The observed down-regulation of the above proteins suggested that glycolysis was inhibited by Cd exposure. According to a previous study, activated immune cells underwent an aerobic glycolytic metabolic transformation and could become potential therapeutic targets for autoimmune diseases [36]. Therefore, these DAPs may become potential targets for treatment strategies for Cd poisoning.

### 4.3. The Effect of Cd Exposure on ABC Transport

Specific transport proteins mediate the transport of specific molecules through lipid membranes. The ABC superfamily is one of the largest transporter protein families. ABC transporters hydrolyze ATP via a nucleotide-binding domain (NBD) to pump the substrate against its electrochemical gradient across the membrane via a transmembrane domain (TMD). They are involved in various physiological processes in different human tissues, and also participate in a variety of cellular processes, such as maintaining osmotic homeostasis, immunity, and lipid transport. Mutations in the relevant ABC transporter genes are involved in the pathogenesis of various human diseases, such as intrahepatic cholestasis. These transporters are also involved in the active pumping of different substrates across the cell membrane. Accordingly, protein-mediated transport regulates the pharmacokinetics of many drugs, and the overexpression of certain transporters in cancer cells is a key element in developing resistance to chemotherapy [42]). In addition, ABC transporters catalyze the transport of toxic compounds out of mammalian cells [43]. According to recent studies, the development and function of T-cell populations were also regulated through ABC transporters [44], providing a promising new avenue for autoimmune therapy, and enhancing immunity against infections and cancer. The metabolomics analysis performed in the current study revealed that the metabolite spermidine involved in ABC transport was down-regulated in the Cd group. Spermidine is a natural polyamine compound that specifically interferes with the tumor cell cycle and triggers autophagy by regulating the key oncological pathways, thereby inhibiting tumor cell proliferation and tumor growth [45]. The down-regulation of spermidine levels indicated that Cd toxicity enhanced the metabolic activity of ABC transport in the black goat, thus weakening the inhibition of cancer occurrence.

### 4.4. Cardiovascular Disease (CVD) and Cd Exposure

CVD is a disease with the highest incidence rate and mortality rate globally, and is the leading cause of global human death [46]. According to previous studies, Cd poisoning induced CVD. Specifically, Cd induced the formation of reactive oxygen species in cardiovascular epithelial cells. As a result, these cells underwent oxidative damage, which eventually led to apoptosis and necrosis, or abnormal lipid metabolism, resulting in cardiovascular injury [47,48]. Further, Cd poisoning in pregnant female animals led to the development of the cardiovascular system after birth [49].

The abundances of *COL1A1* and *GAPDH*, two proteins related to human diseases, were altered by Cd exposure in the current study. *COL1A1* is closely related to the occurrence of malignant tumors [50]. It is highly expressed in various cancers and regulates various processes, such as cell proliferation, metastasis, and apoptosis. *GAPDH* is one of the most important glycolytic enzymes. It catalyzes the sixth step of glycolysis and produces NADH [51]. Glycolysis has a protective effect on DNA damage, and previous research has confirmed that glycolytic inhibitors may improve the outcomes of cancer treatment [51]. The down-regulation of these two proteins related to CVD suggested that Cd exposure might cause cardiovascular system damage in the goat.

## 5. Conclusions

In the current study, a combined proteomic and metabolomic analysis indicated that Cd exposure affected diverse biological processes in the goat. Thirty metabolites identified in the goat serum in relation to Cd poisoning were mainly related to arginine and proline metabolism, beta-alanine metabolism, and glutathione metabolism. Further, the abundances of 108 proteins were affected in goats from the Cd group. Bioinformatics analysis elucidating the mechanism of Cd toxicity revealed several critical down-regulated DAPs, such as *COL1A1* and *GAPDH* involved in CVD, in association with Cd poisoning. *CKM* might be a potential protein marker, and spermidine might be a potential metabolic marker of Cd poisoning in goats. The current study demonstrated that the combination of proteomics and metabolomics could be used to inform the approaches for the early diagnosis of Cd poisoning in animals.

## Figures and Tables

**Table 1:** Basal diet composition and nutrient levels (%).

Ingredient	Content	Nutrient Component(s) ②	Content
Corn	32.00	ME/(MJ/kg)	9.80
Peanut shell	8.00	DM,%	88.20
Corn straw	18.70	CP,%	14.75
Fermented rapeseed meal	14.00	EE,%	3.20
Soybean meal	6.55	NDF,%	20.40
Wheat bran	10.00	Ca,%	0.90
Calcium carbonate	0.90	P,%	0.60
Calcium hydrogen phosphate	0.85	Cd, mg·kg^−1^	0.12
NaCl	5.00	-	-
Compound premix ①	4.00	-	-
Total	100.00	-	-

① Provide per kg of feed: VA 100 000 IU, VD3 12 000 IU, VE 950 IU, Cu 15 mg, Fe 50 mg, Zn 30 mg, Mn 40 mg, Co 0.15 mg, Mo 0.50 mg, and Se 0.20 mg. ② Measured values: DM, CP, EE, Ca, P, and Cd. Calculated values: ME and NDF.

**Table 2:** Serum metabolites with a significantly altered abundance in goats upon Cd exposure, based on OPLS-DA.

No.	RT (min)	*m*/*z*	Formula	Metabolites	VIP	Change	Fold Change	*p*-Value
1	6.203	585.27034	C_33_H_36_N_4_O_6_	(3Z)-Phytochromobilin	3.29	Down	0.27	0.04283
2	4.86	419.13565	C_21_H_24_O_9_	Davidioside	2.40	Down	0.48	0.02233
3	8.465	323.22314	C_19_H_32_O_4_	Decylubiquinol	2.11	Down	0.62	0.00964
4	7.401	288.28959	C_17_H_37_NO_2_	2,2′-(Tridecylimino)diethanol	1.03	Down	0.89	0.03681
5	0.104	284.29459	C_18_H_37_NO	Stearamide	1.26	Down	0.86	0.00261
6	7.775	279.19698	C_17_H_28_O_3_	Methyl(2E_6E)-(10R_11S)-10_11-epoxy-3_7_11-trimethyltrideca-2_6-dienoate	2.70	Down	0.40	0.03969
7	6.789	263.16431	C_6_H_12_N_2_	DABCO	2.27	Down	0.54	0.02288
8	5.676	253.13489	C_16_H_18_N_2_O	Elymoclavine	2.36	Down	0.44	0.03908
9	4.146	214.12659	C_11_H_19_NOS	Octhilinone	2.58	Down	0.49	0.01609
10	0.498	195.12285	C_8_H_18_O_5_	PEG-4	1.24	Down	0.88	0.00002
11	0.463	181.12245	C_11_H_16_O_2_	2-Tert-butyl-4-methoxyphenol	1.05	Down	0.91	0.00009
12	0.371	175.09659	C_8_H_14_O_4_	Suberic acid	1.17	Down	0.88	0.00055
13	0.198	172.13338	C_9_H_17_NO_2_	Gabapentin	1.08	Down	0.90	0.00114
14	0.53	170.11764	C_9_H_15_NO_2_	Diacetone acrylamide	1.03	Down	0.91	0.00061
15	0.453	162.04151	C_6_H_5_N_5_O	Pterin	2.93	Down	0.38	0.02406
16	0.332	151.09666	C_6_H_14_O_4_	Triethylene glycol	1.20	Down	0.88	0.00013
17	4.899	149.04503	C_5_H_10_O_6_	D-Arabinonate	2.63	Down	0.51	0.01107
18	0.814	146.16534	C_7_H_19_N_3_	Spermidine	2.94	Down	0.47	0.01647
19	0.211	133.08609	C_6_H_12_O_3_	2-Hydroxycaproic acid	1.09	Down	0.90	0.00018
20	0.233	110.06049	C_6_H_7_NO	4-Aminophenol	1.13	Down	0.90	0.00000
21	0.292	107.0707	C_4_H_10_O_3_	Diethylene glycol	1.08	Down	0.89	0.00230
22	11.424	338.34145	C_22_H_43_NO	Erucic amide	2.20	Up	1.85	0.02459
23	6.315	331.11526	C_8_H_10_O_3_	Hexahydrophthalic anhydride	1.82	Up	1.64	0.04384
24	11.935	311.29601	C_20_H_40_O_2_	Eicosanoic acid	1.81	Up	1.44	0.00931
25	6.311	307.11926	C_17_H_16_N_4_O_2_	Nifenazone	2.18	Up	1.81	0.02808
26	11.186	293.17603	C_17_H_26_O_4_	[6]-Gingerol	2.14	Up	1.57	0.01390
27	1.115	261.03212	C_15_H_10_O_2_	Flavone	2.00	Up	1.57	0.01417
28	0.069	187.00646	C_7_H_8_O_4_S	p-Cresol sulfate potassium; p-Cresol sulfate	1.81	Up	1.42	0.02095
29	10.042	136.02156	C_7_H_5_NS	1,3-Benzothiazole	1.02	Up	1.11	0.01268
30	0.454	103.03903	C_4_H_8_O_3_	3-Hydroxybutyric acid	1.47	Up	1.28	0.01322

RT: retention time.

**Table 3:** The top 10 up- and down-regulated proteins in the Cd group.

Regulate	Accession	Gene Name	Protein Description	Number of Peptides	Number of Unique Peptides	MW (kDa)	Score	Coverage (%)	Ratio	*p*-Value
Down	A0A452ED36	*BCAP31*	B-cell receptor-associated protein	1	1	29.1	0	3.54	0.14	7.42 × 10^−7^
Down	A0A452FZ73	*CKM*	Creatine kinase	6	5	43	46.51	17.85	0.24	2.34 × 10^−7^
Down	A0A452E132	*PGK1*	Phosphoglycerate kinase	3	3	44.4	35.99	10.36	0.25	4.73 × 10^−7^
Down	A0A452FR97	*PGAM1*	Phosphoglycerate mutase	2	2	28.9	32.76	9.84	0.28	4.04 × 10^−6^
Down	A0A8C2NM51	-	Tropomyosin 1	1	1	11.6	0	8.65	0.28	2.00 × 10^−7^
Down	A0A8C2RRP4	-	Trio Rho guanine nucleotide exchange factor	1	1	205.4	0	0.28	0.32	3.02 × 10^−5^
Down	A0A8C2S7B0	*ALDOA*	Fructose-bisphosphate aldolase	6	5	39.2	44.47	24.38	0.38	1.26 × 10^−5^
Down	A0A452FNW6	*GAPDH*	Glyceraldehyde-3-phosphate dehydrogenase	7	2	35.9	377.03	21.02	0.4	7.55 × 10^−6^
Down	A0A452G1C7	-	DnaJ heat shock protein family (Hsp40) member A1	1	1	40.2	0	1.68	0.41	5.82 × 10^−6^
Down	A0A452FAN0	*ENO3*	Phosphopyruvate hydratase	2	1	47	141.08	4.61	0.42	4.97 × 10^−6^
Up	A0A452DSW4	*LOC102168295*	Amine oxidase	11	2	84.3	1066.98	14.02	1.57	0
Up	A0A452EH87	-	Ig-like domain-containing protein	2	2	12.41	330.74	19.49	1.59	0
Up	A0A452FEE2	-	Ig-like domain-containing protein	1	1	15.05	44.34	10.42	1.6	0.001
Up	A0A8C2RPZ7	-	Family with sequence similarity 180 member B	1	1	19.49	0	23.86	1.64	0.004
Up	A0A452ECK4	*CDH17*	Cadherin 17	1	1	92.36	0	1.92	1.68	0
Up	A0A8C2QUL2	*KANSL3*	KAT8 regulatory NSL complex subunit 3	1	1	88.65	0	1.65	1.71	2.44 × 10^−5^
Up	A0A452FQB9	*-*	Ig-like domain-containing protein	2	2	41.82	48.76	5.66	1.95	3.20 × 10^−5^
Up	A0A452DYK6	*DNAAF11*	Dynein axonemal assembly factor 11	1	1	54.8	0	2.96	2.08	0.001
Up	A0A452DWC1	*CFAP52*	Cilia and flagella associated protein 52	1	1	68.65	0	1.61	2.09	2.55 × 10^−5^
Up	A0A452G904	*NRCAM*	Neuronal cell adhesion molecule	1	1	129.05	0	0.52	5.67	2.92 × 10^−7^

## Data Availability

The datasets generated during and/or analyzed during the current study are available from the corresponding author on reasonable request.

## Supplementary Materials

The following supporting information can be downloaded at: https://www.mdpi.com/article/10.3390/ani13172705/s1, Figure S1, Flowchart of the metabolomics and proteomics experiments; Figure S2, The representative total ion chromatograms of the serum sample analysed by the HILIC UHPLC-Q-EXACTIVE/MS; Figure S3, PCA score plots derived from HILIC UHPLC-Q-EXACTIVE/MS in positive (a) and negative (b) ionization modes; Figure S4, PLS-DA score plots derived from HILIC UHPLC-Q-Exactive/MS in positive (a) and negative (b) ionization modes; Figure S5, Bubble diagram of top-10 KEGG enrichment results according to their *p*-values; Figure S6, Gene Ontology; Figure S7, Network of significant altered metabolic pathways in response to Cd poisoning in goats and the correlated thermogram analysis of proteins and metabolites. Table S1, Detailed information of metabolites; Table S2, The results of Compound Discoverer pathway analysis; Table S3, Detailed information of proteins; Table S4, Enriched KEGG pathways of differentially abundant proteins in comparision with the control group; Table S5, Confirmation of DAPs detected using parallel reaction monitoring and TMT analysis. Detailed verification procedures were described in the Supplementary File S1.
