# Supplementary material for: Cadmium Exposure Affects Serum Metabolites and Proteins in the Male Guizhou Black Goat
Source: Animals (Basel). 2023 Aug 25;13(17):2705. doi: 10.3390/ani13172705 (PMC10487163; doi:10.3390/ani13172705)
Supplement: Supplementary file 1 [file animals-13-02705-s001.zip › Supplement Tables and Figures.pdf]

Figure S1. Flowchart of the metabolomics and proteomics experiments

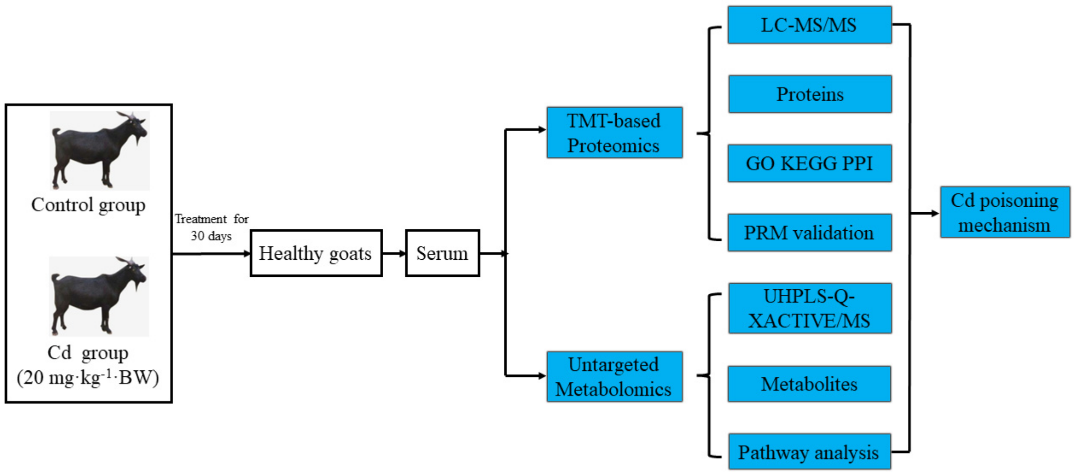

Figure S2. The representative total ion chromatograms of the serum sample analysed by the HILIC UHPLC-Q-EXACTIVE/MS. (a): Quality control (QC); (b): the control group; (c): Cd group.

(a)

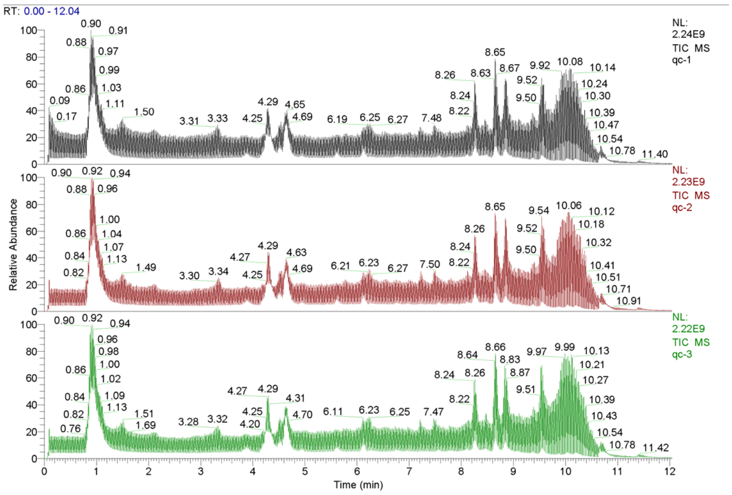

(b)

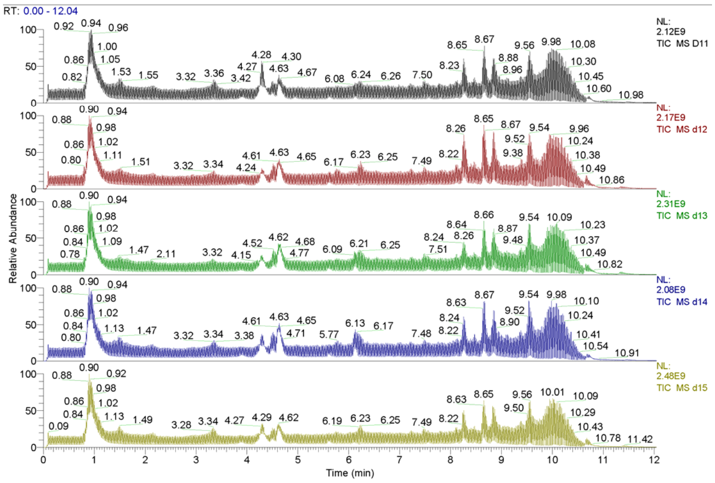

(c)

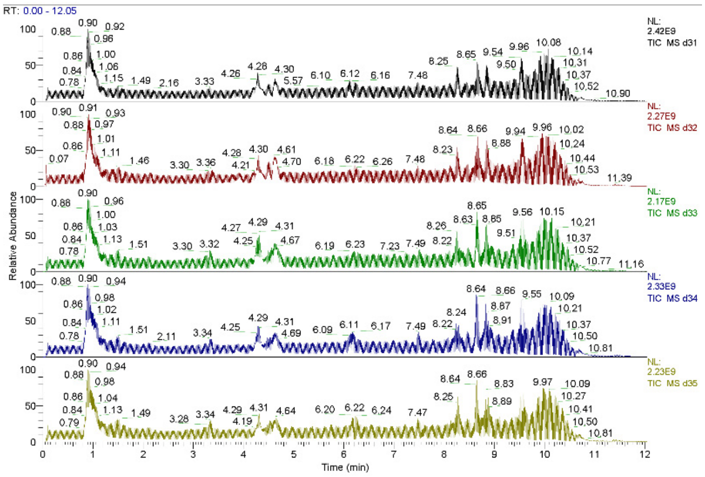

Figure S3. PCA score plots derived from HILIC UHPLC-Q-EXACTIVE/MS in positive (a) and negative (b) ionization modes.

(a)

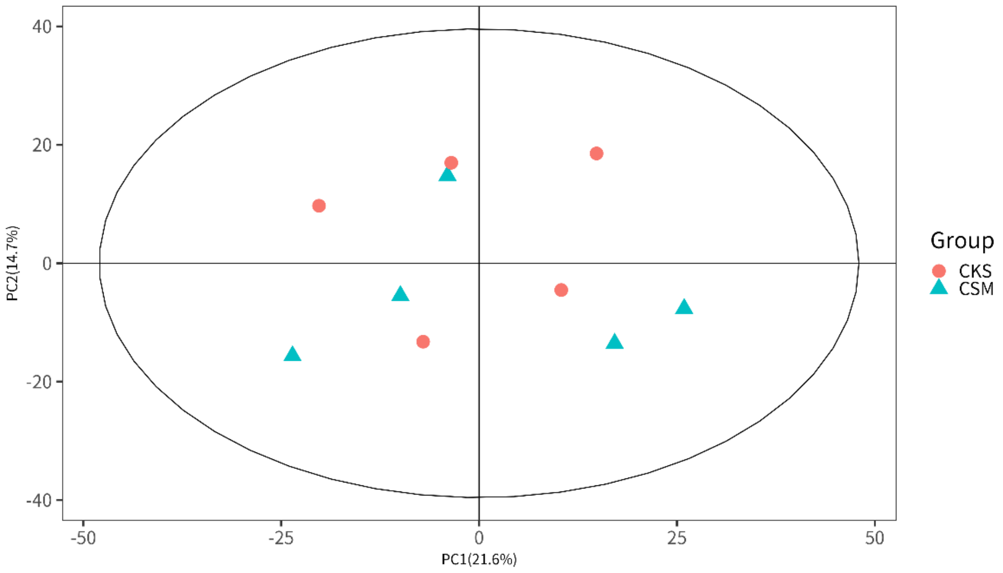

(b)

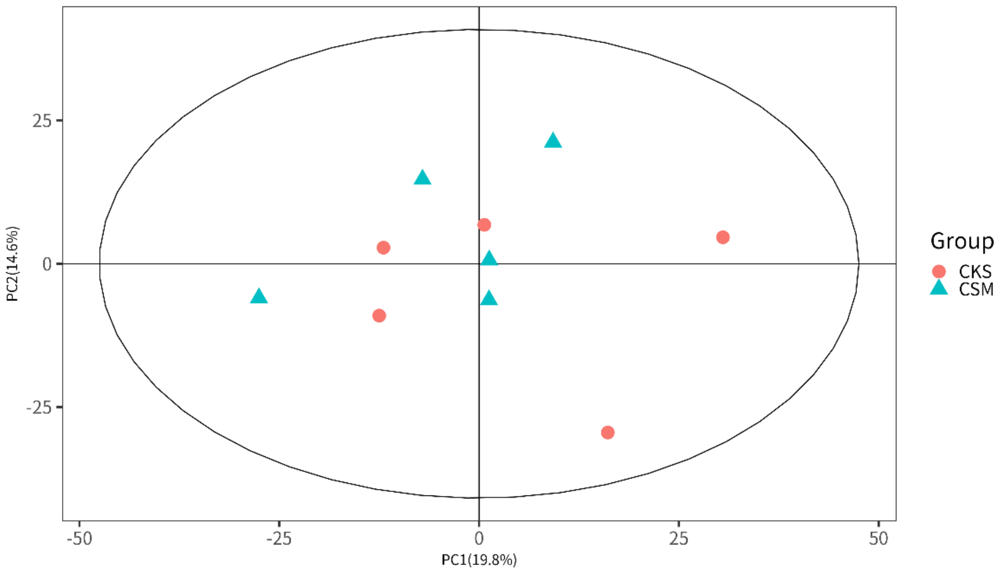

Figure S4. PLS-DA score plots derived from HILIC UHPLC-Q-Exactive/MS in positive (a) and negative (b) ionization modes.

(a)

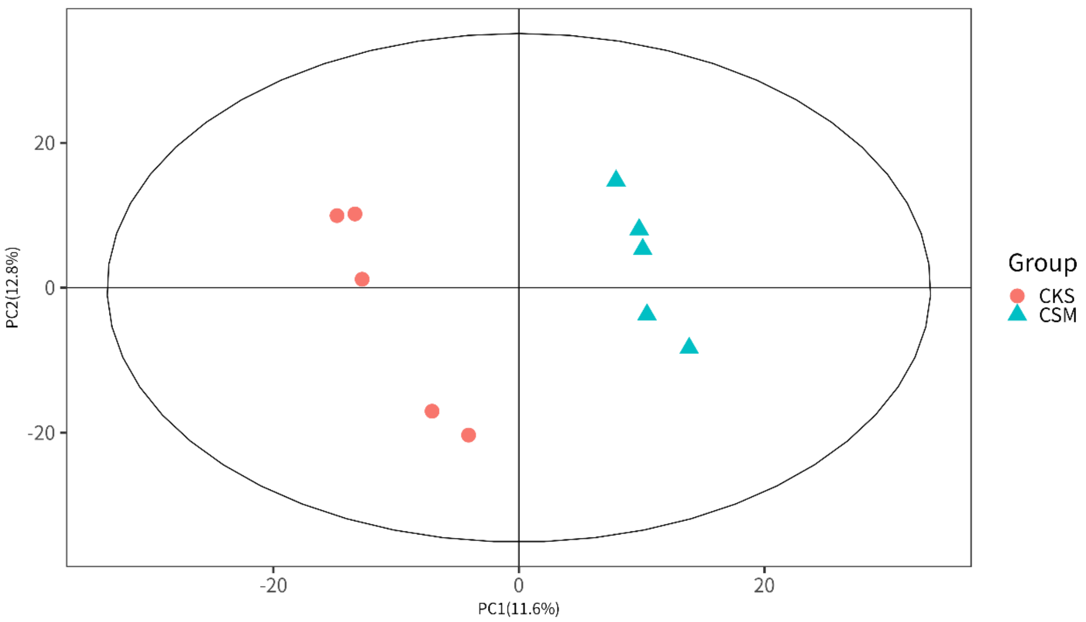

(b)

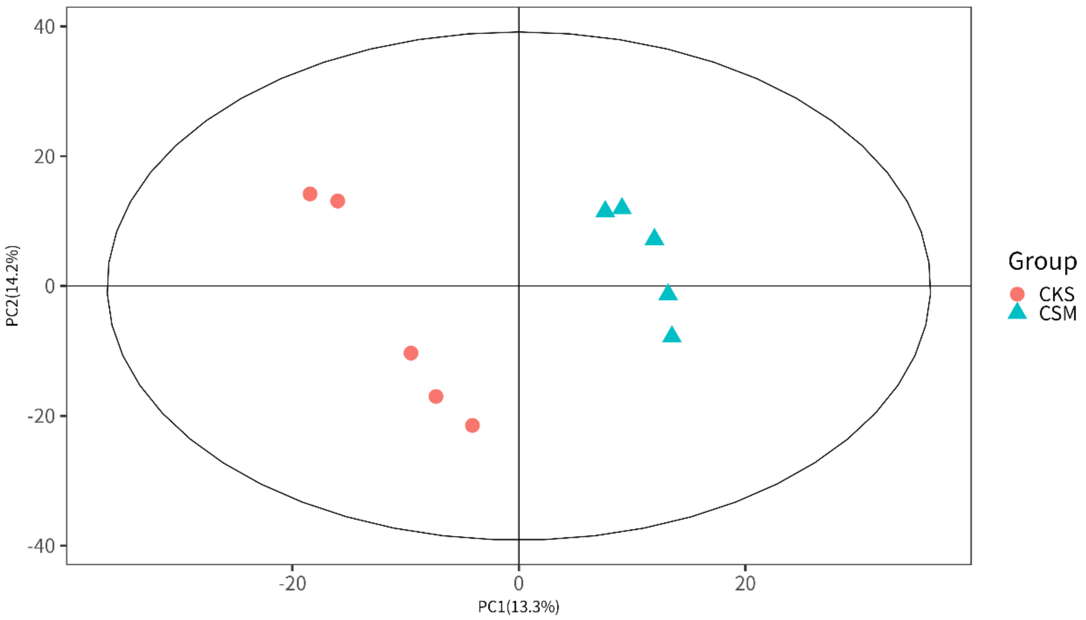

Figure S5. Bubble diagram of top-10 KEGG enrichment results according to their P-values.

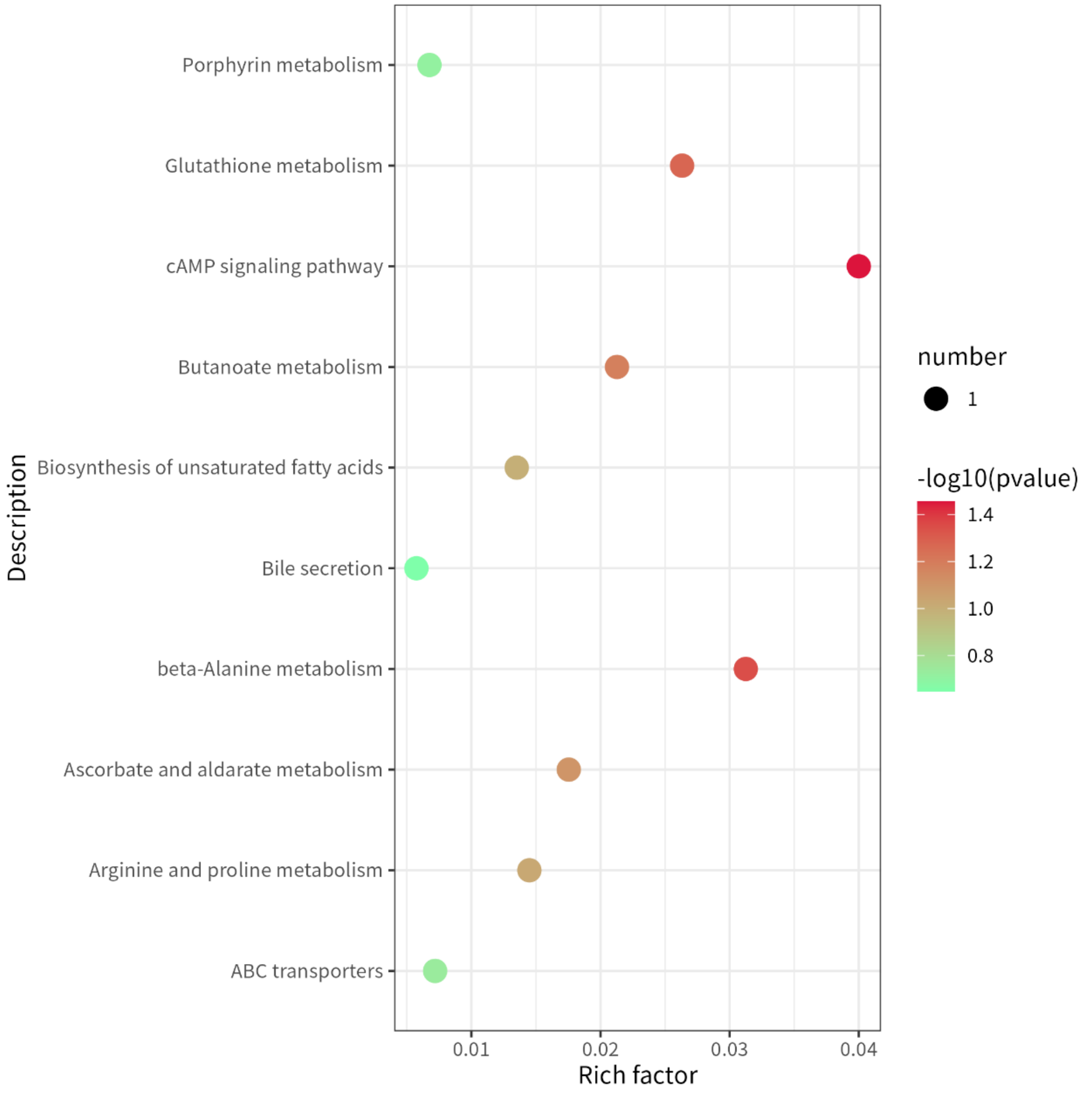

Figure S6. Gene Ontology. (a): Cellular compartments, CC; (b): Molecular function, MF; (c): Biological process, BP.

(a)

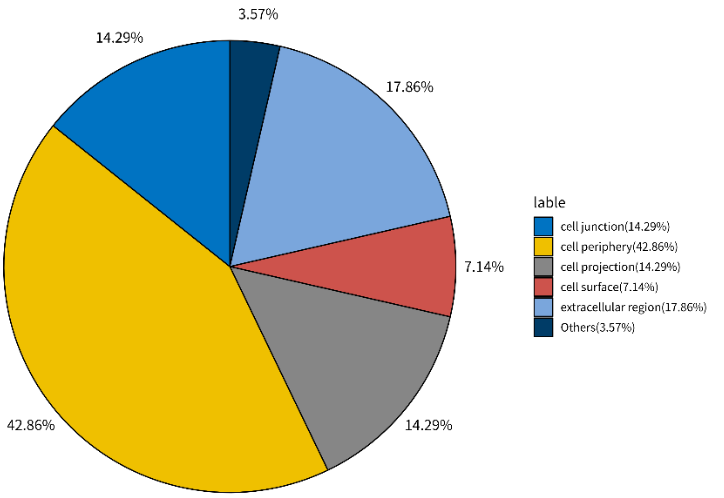

(b)

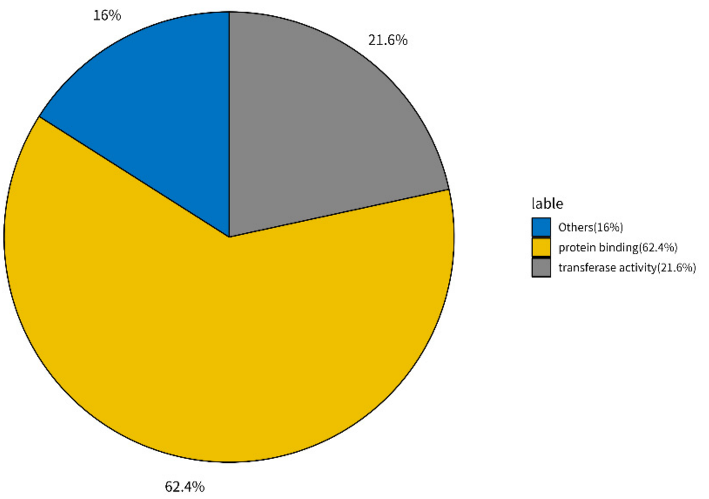

(c)

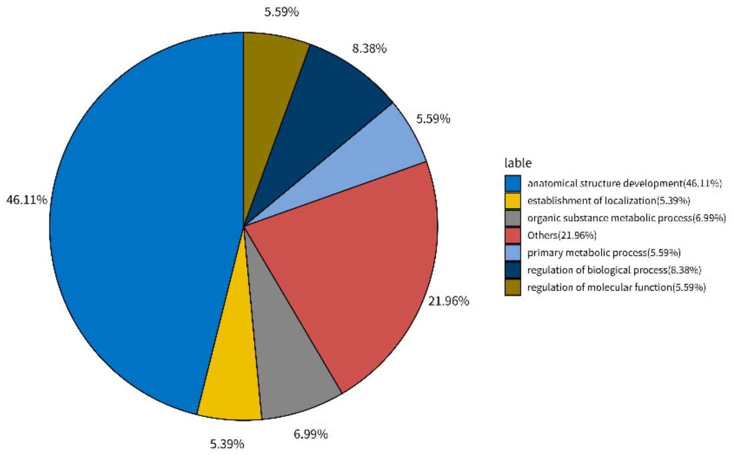

Figure S7. (a) Network of significant altered metabolic pathways in response to Cd poisoning in goats. According to KEGG, with the integration of proteomics and metabolomics, the network for mechanism explanation was established. Metabolite–protein interaction network based on the differential metabolites and DEPs for ESBL-EC vs. non-ESBL-EC in positive mode. The squares represent differential metabolites. The circles represent differential protein genes. The red/green box represents the protein mapped to the same pathway, and the blue /yellow circle represents the metabolite mapped to the same pathway, where red/ yellow represents the up-regulated protein/metabolite, and green/blue represents the down-regulated protein / metabolite.

(b) The correlated thermogram analysis of proteins and metabolites. Each row represents a differential protein and each column represents a differential metabolite. Each cell represents a protein associated with a metabolite, including correlation coefficient corr and correlation p value (pvalue) information. The value range of correlation coefficient corr is (- 1, + 1). In the figure, it is represented by color, corr > 0 indicates positive correlation, and it is represented by red; corr < 0 indicates negative correlation, and blue indicates that the darker the color, the greater the correlation; corr = 0 indicates that there is no correlation, which is represented by yellow. P value reflects the significant level of correlation. P value < 0.05 indicates that there is a significant correlation between protein and metabolites, expressed by "*".

(a)

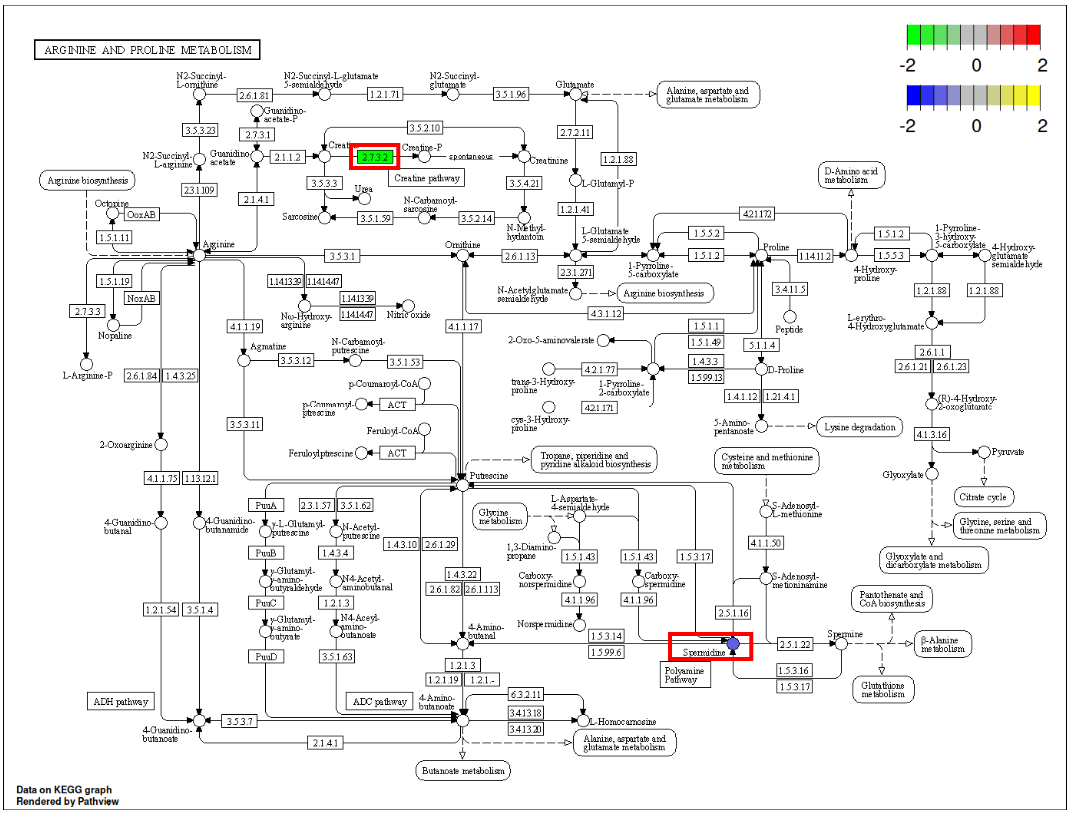

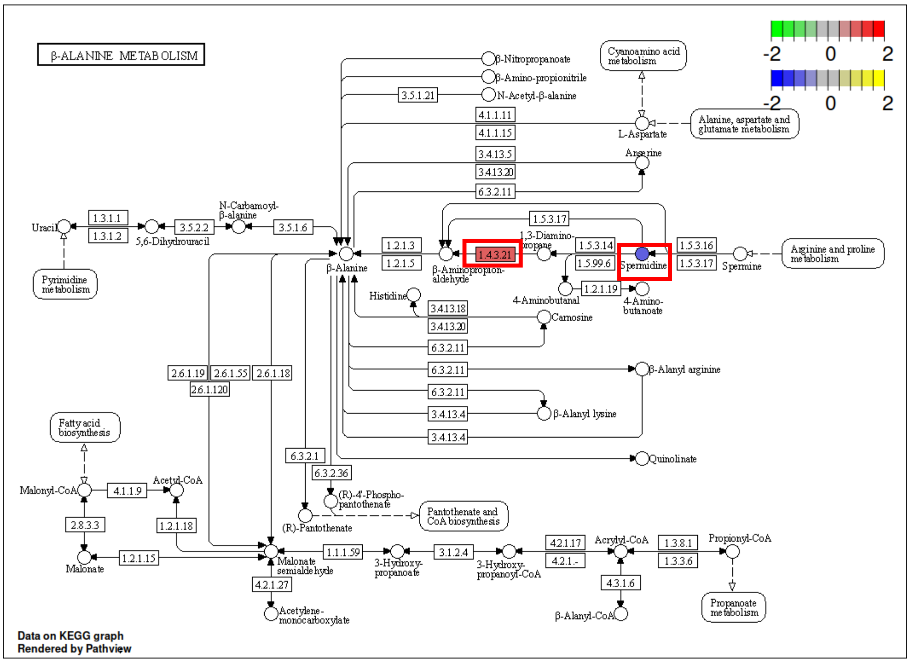

β-ALANINE METABOLISM
-2 0 2
-2 0 2
β-Nitropropanoate
β-Amino-propionitrile
N-Acetyl-β-alanine
Cyanoamino acid metabolism
Alanine, aspartate and glutamate metabolism
L-Aspartate
Anserine
3.5.1.21
4.1.1.11
4.1.1.15
3.4.13.5
3.4.13.20
6.3.2.11
1.3.1.1
1.3.1.2
3.5.2.2
3.5.1.6
N-Carbamoyl-β-alanine
Uracil
5,6-Dihydrouracil
Pyrimidine metabolism
1.2.1.3
1.2.1.5
β-Alanine
1.4.3.21
β-Aminopropion-aldehyde
1.3-Diamino-propane
1.5.3.17
1.5.3.14
1.5.99.6
Spermidine
1.5.3.16
1.5.3.17
Spermine
Arginine and proline metabolism
4-Aminobutanal
1.2.1.19
4-Amino-butanoate
Histidine
3.4.13.18
3.4.13.20
Carnosine
6.3.2.11
β-Alanyl arginine
β-Alanyl lysine
3.4.13.4
Quinolinate
2.6.1.19
2.6.1.55
2.6.1.18
2.6.1.120
Fatty acid biosynthesis
Malonyl-CoA
4.1.1.9
Acetyl-CoA
2.8.3.3
1.2.1.18
Malonate
1.2.1.15
6.3.2.1
6.3.2.36
(R)-4'-Phospho-pantothenate
Pantothenate and CoA biosynthesis
(R)-Pantothenate
Malonate semialdehyde
4.2.1.27
Acetylene-monocarboxylate
1.1.1.59
3-Hydroxy-propanoate
3.1.2.4
3-Hydroxy-propanoyl-CoA
4.2.1.17
4.2.1.-
Acrylyl-CoA
1.3.8.1
1.3.3.6
Propionyl-CoA
4.3.1.6
β-Alanyl-CoA
Propanoate metabolism
Data on KEGG graph
Rendered by Pathview

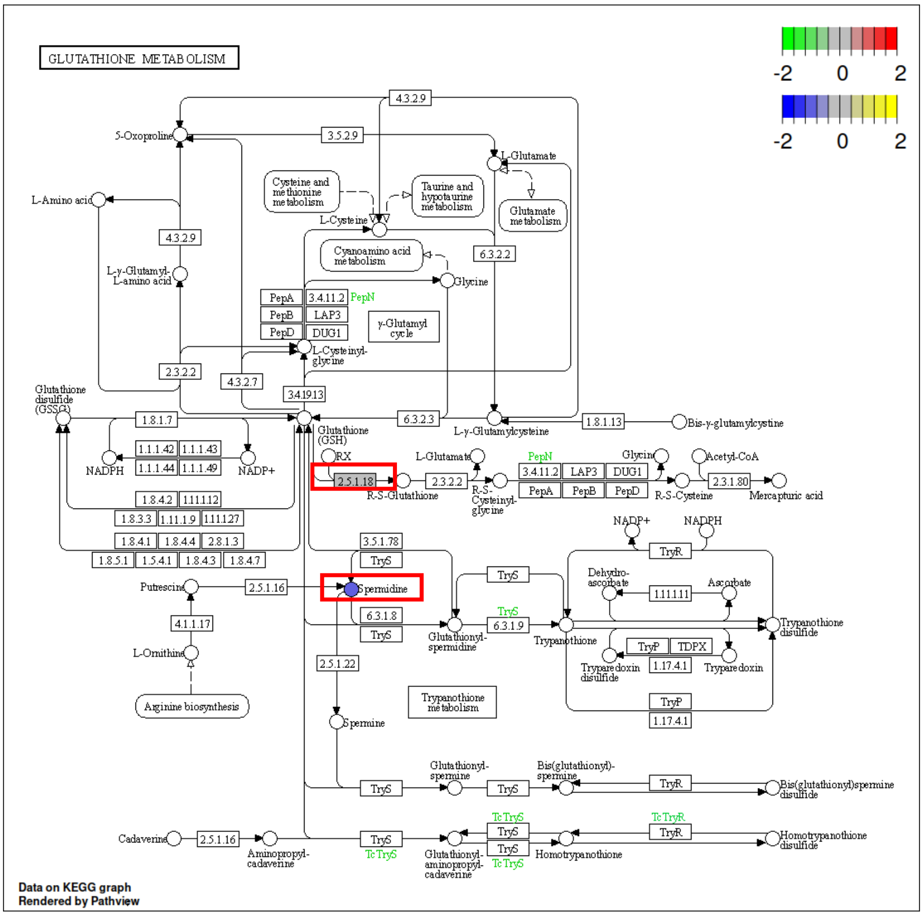

GLUTATHIONE METABOLISM
-2 0 2
-2 0 2
4.3.2.9
3.5.2.9
5-Oxoproline
L-Glutamate
L-Amino acid
Cysteine and methionine metabolism
Taurine and hypotaurine metabolism
Glutamate metabolism
L-Cysteine
4.3.2.9
L-γ-Glutamyl-L-amino acid
Cyanoamino acid metabolism
6.3.2.2
Glycine
PepA
3.4.11.2
PepN
PepB
LAP3
PepD
DUG1
γ-Glutamyl cycle
L-Cysteinyl-glycine
2.3.2.2
4.3.2.7
3.4.19.13
Glutathione disulfide (GSSG)
1.8.1.7
6.3.2.3
L-γ-Glutamylcysteine
1.8.1.13
Bis-γ-glutamylcystine
Glutathione (GSH)
RX
L-Glutamate
Glycine
Acetyl-CoA
NADPH
1.11.1.42
1.11.1.43
1.11.1.44
1.11.1.49
NADP+
2.5.1.18
R-S-Glutathione
2.3.2.2
R-S-Cysteinyl-glycine
3.4.11.2
LAP3
DUG1
PepA
PepB
PepD
R-S-Cysteine
2.3.1.80
Mercapturic acid
1.8.4.2
1.11.1.12
1.8.3.3
1.11.1.9
1.11.1.27
1.8.4.1
1.8.4.4
2.8.1.3
1.8.5.1
1.5.4.1
1.8.4.3
1.8.4.7
3.5.1.78
TryS
NADP+
NADPH
TryR
Putrescine
2.5.1.16
Spermidine
Dehydro-ascorbate
1.11.1.11
Ascorbate
6.3.1.8
6.3.1.9
4.1.1.17
L-Ornithine
Glutathionyl-spermidine
Trypanothione
Trypanothione disulfide
TryP
TDPX
Tryparedoxin disulfide
1.17.4.1
Tryparedoxin
2.5.1.22
Arginine biosynthesis
Trypanothione metabolism
Spermine
Glutathionyl-spermine
Bis(glutathionyl)-spermine
Bis(glutathionyl)spermine disulfide
Cadaverine
2.5.1.16
Aminopropyl-cadaverine
TcTryS
Glutathionyl-aminopropyl-cadaverine
Homotrypanothione
TcTryR
Homotrypanothione disulfide
Data on KEGG graph
Rendered by Pathview

(b)

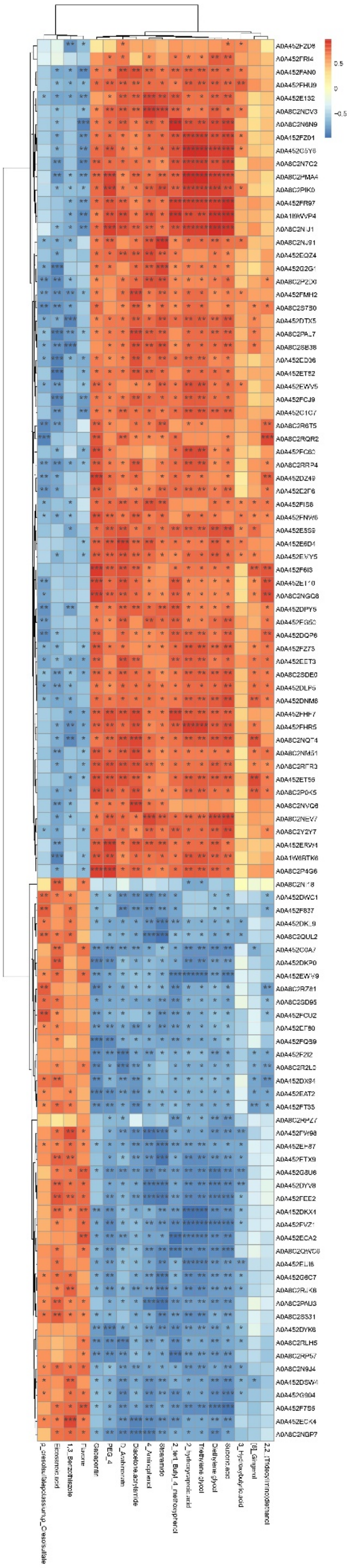

Table S4. Enriched KEGG pathways of differentially abundant proteins in comparision with the control group.

Pathway ID	Pathway name	Proteins	Count	P-value_adjusted
chx00360	Phenylalanine metabolism	PAO|2	2	0.014
chx00410	beta-Alanine metabolism	PAO|2	2	0.014
chx00350	Tyrosine metabolism	PAO|2	2	0.014
chx00260	Glycine, serine and threonine metabolism	PAO|2	2	0.014
chx05226	Gastric cancer	CDH17|1;WNT10B|1	2	0.113
chx05225	Hepatocellular carcinoma	GSTA2|1;WNT10B|1	2	0.124
chx05205	Proteoglycans in cancer	COL1A1|1;WNT10B|1	2	0.137
chx05415	Diabetic cardiomyopathy	COL1A1|1;GAPDH|1	2	0.137
chx04979	Cholesterol metabolism	APOH|1	1	0.192
chx05165	Human papillomavirus infection	COL1A1|1;WNT10B|1	2	0.192
chx00330	Arginine and proline metabolism	CKM|1	1	0.192
chx05010	Alzheimer disease	GAPDH|1;WNT10B|1	2	0.192
chx00480	Glutathione metabolism	GSTA2|1	1	0.192
chx05217	Basal cell carcinoma	WNT10B|1	1	0.192
chx00010	Glycolysis / Gluconeogenesis	GAPDH|1	1	0.192
chx00982	Drug metabolism - cytochrome P450	GSTA2|1	1	0.192
chx05204	Chemical carcinogenesis - DNA adducts	GSTA2|1	1	0.192

chx00980	Metabolism of xenobiotics by cytochrome P450	GSTA2|1	1	0.192
chx01230	Biosynthesis of amino acids	GAPDH|1	1	0.192
chx01524	Platinum drug resistance	GSTA2|1	1	0.192
chx00983	Drug metabolism - other enzymes	GSTA2|1	1	0.192
chx03008	Ribosome biogenesis in eukaryotes	NXT2|1	1	0.192
chx04512	ECM-receptor interaction	COL1A1|1	1	0.196
chx03015	mRNA surveillance pathway	NXT2|1	1	0.196
chx04916	Melanogenesis	WNT10B|1	1	0.196
chx04933	AGE-RAGE signaling pathway in diabetic	COL1A1|1	1	0.196
chx04066	HIF-1 signaling pathway	GAPDH|1	1	0.196
chx05146	Amoebiasis	COL1A1|1	1	0.196
chx03013	Nucleocytoplasmic transport	NXT2|1	1	0.196
chx01200	Carbon metabolism	GAPDH|1	1	0.196
chx04611	Platelet activation	COL1A1|1	1	0.202
chx04926	Relaxin signaling pathway	COL1A1|1	1	0.202
chx04974	Protein digestion and absorption	COL1A1|1	1	0.202
chx04550	Signaling pathways regulating pluripotency of	WNT10B|1	1	0.202
chx05418	Fluid shear stress and atherosclerosis	GSTA2|1	1	0.202
chx05224	Breast cancer	WNT10B|1	1	0.202

chx04934	Cushing syndrome	WNT10B|1	1	0.202
chx04390	Hippo signaling pathway	WNT10B|1	1	0.202
chx04150	mTOR signaling pathway	WNT10B|1	1	0.202
chx04310	Wnt signaling pathway	WNT10B|1	1	0.209
chx05164	Influenza A	NXT2|1	1	0.209
chx04510	Focal adhesion	COL1A1|1	1	0.236
chx05207	Chemical carcinogenesis - receptor activation	GSTA2|1	1	0.246
chx05208	Chemical carcinogenesis - reactive oxygen species	GSTA2|1	1	0.257
chx05132	Salmonella infection	GAPDH|1	1	0.273
chx04151	PI3K-Akt signaling pathway	COL1A1|1	1	0.354
chx05014	Amyotrophic lateral sclerosis	NXT2|1	1	0.354
chx05022	Pathways of neurodegeneration - multiple diseases	WNT10B|1	1	0.422

Table S5. Confirmation of DAPs detected using parallel reaction monitoring and TMT analysis

Protein name	Description	Gene	TMT results	PRM results
A0A452G0A7	Amine oxidase (EC 1.4.3.-)	SERPINA7	1.20	3.52
A0A452DYV8	Alpha-2-glycoprotein 1, zinc-binding	-	1.36	2.82
A0A452EH87	Ig-like domain-containing protein	C5	1.59	3.41
A0A452ETX9	Alpha-2-macroglobulin	-	1.34	3.46
A0A452F7S5	Beta-2-microglobulin	VNN1	1.26	1.29
A0A452FEE2	Ig-like domain-containing protein	LOC102168547<br>B2M	1.60	5.42
A0A452G6C7	Ig-like domain-containing protein	LOC102176354	1.41	2.56
