# Supplementary material for: Cadmium Exposure Affects Serum Metabolites and Proteins in the Male Guizhou Black Goat
Source: Animals (Basel). 2023 Aug 25;13(17):2705. doi: 10.3390/ani13172705 (PMC10487163; doi:10.3390/ani13172705)
Supplement: Supplementary file 1 [file animals-13-02705-s001.zip › Supplementary file S1.pdf]

Serum preparation for metabolomics

The serum samples were ground into powder with liquid nitrogen, and added into 400 μL precooled formic acid and acetonitrile in aqueous solution at 4 °C (methanol: acetonitrile: water = 4:4:2, v/v), vortex-mixed for 1 min. The mixture was incubated for 60 min at -20 °C and centrifuged at 14,000g for 20 min at 4 °C to precipitate the proteins. Next, the supernatant was filtered through a 0.22 μm nylon filter, and an aliquot of 2μL was injected for HILIC UHPLC-Q-EXACTIVE/MS analysis.

Metabolomics data acquisition

The optimal linear gradient program was shown as followed: 0–0.5min, 5% B; 0.5–1.0 min, 5% B; 1.0–9.0 min, 5–100% B; 9.0–12.0 min, 100% B; 12.0–15.0 min, 5% B. Post time was set to 3 min for equilibration.

The scan time was set at 5 spectra/s and the data was collected in centroid mode from 50 to 1000 m/z. The optimal conditions of analysis were as followed: desolvation gas rate was set to 10 L/min at 320 °C and the nebulizer pressure was set at 60 psig; the fragment voltage was 60 V in both modes, capillary voltages were 3.5 KV in positive and negative modes.

NanoLC-PRM analyses

NanoLC-PRM analyses were performed on a NanoAcquity UPLC device (Waters, Milford, Massachusetts, USA) coupled to a Q-Exactive HF (Thermo Fisher Scientific, USA). Solvent system consisted of 0.1% formic acid (FA) in H_2O (solvent A) and 0.1% FA in ACN (solvent B) (Optima LC/MS grade solvents, Fisher Chemical, Illkirch, France). Peptides (100 ug) were separated onto a Home-made C18 column (20 mm ×

100 μm, 5 μm diameter particles) over 3 min at 250 μL/min with 1% solvent B. Peptides were eluted on a EASY C18 column (120 mm × 75 μm, 1.90 μm particles; Thermo scientific, USA) at 0.25 μL/min with the following gradient of solvent B: from 5% to 23% in 0-42 min, linear from 23% to 40% in 42-50 min, the linear gradient of liquid B in 52-60 min rises to 100% and maintains. A scheduled PRM method consisting of one full MS1 scan and 20 targeted MS2 scans was developed. The full MS1 scan was collected from 300 to 1800 m/z at a resolution of 60,000 at 200 m/z (AGC target: 3e6, maximum IT: 200 ms). Targeted MS2 scans were collected at a resolution of 30,000 at 200 m/z (AGC target: 1e6, maximum IT: 120 ms) and scheduled with 1.6Th isolation window. Precursors were isolated within a 2-m/z window and fragmented with a normalized collision energy (NCE) of 27.

Multivariate statistical analysis

The obtained data sets were introduced into SIMCA-P 14.1 (Umetrics，Umea，Sweden) to perform unsupervised principal component analysis (PCA), supervised partial least squares discriminant analysis (PLS-DA), and supervised orthogonal partial least squares discriminant analysis (OPLS-DA). The fitness and predictive capability of the PCA, PLS-DA, and OPLS-DA models were evaluated by the cumulative R^2 and Q^2. Variable importance for projection (VIP) value and loading-plots were constructed from the OPLS. Student's t-test with false discovery rate (FDR) was employed for univariate analysis across groups. The R language software was used for one-dimensional statistical analysis such as volcano map analysis, Student's t-test, and multiple of variation analysis to find the differential metabolites

related to Cd poisoning.

Quantitative proteomic analysis

A linear gradient program was shown as followed: 0–35% buffer B for 50 min, 35–100% buffer B for 8 min, hold in 100% buffer B for 2 min. LC-MS/MS analysis was performed on a Q-EXACTIVE MS (Thermo Fisher Scientific). The mass spectrometer was operated in positive ion mode. MS data was acquired using a data-dependent top 10 method dynamically choosing the most abundant precursor ions from the survey scan (300–1800 m/z) for higher energy collisional dissociation (HCD) fragmentation. Automatic gain control (AGC) target was set to 3e6, and maximum inject time to 10 ms. Dynamic exclusion duration was 40.0 s. Survey scans were acquired at a resolution of 70,000 at m/z 200 and resolution for HCD spectra was set to 17,500 at m/z 200, and isolation width was 2 m/z. Normalized collision energy was 30 eV and the underfill ratio was defined as 0.1%.
